# Exploring the Antifungal, Antibiofilm, and Wound Healing In Vitro Properties of *N*-(4-Methoxycinnamoyl)-Anthranilic Acid as a Supportive Strategy for Ocular Fungal Infections

**DOI:** 10.3390/antibiotics15060597

**Published:** 2026-06-11

**Authors:** Francesco Petrillo, Annalisa Buonanno, Angela Maione, Luigi Longobardo, Michele Reibaldi, Emilia Galdiero, Armando Zarrelli, Marco Guida

**Affiliations:** 1 Department of Ophthalmology, “City of Health and Science” Hospital, 10126 Turin, Italy; francesco.petrillo@aocardarelli.it (F.P.); michele.reibaldi@unito.it (M.R.); 2 Department of Biology, University of Naples Federico II, 80126 Naples, Italy; annalisa.buonanno@unina.it (A.B.); egaldier@unina.it (E.G.); marco.guida@unina.it (M.G.); 3 Department of Chemical Science, University of Napoli Federico II, Via Cinthia 4, 80126 Napoli, Italy; luilongo@unina.it

**Keywords:** green synthesis, antifungal activity, human cells, *Candidozyma auris*, *Candida albicans*, keratitis

## Abstract

**Background**: Fungal ocular infections, including keratitis and endophthalmitis, remain difficult to treat due to limited antifungal efficacy, poor tissue penetration, and biofilm-mediated resistance. This study evaluated the antifungal and host-protective potential of *N*-(4-methoxycinnamoyl)-anthranilic acid (NMCA) against *Candida albicans* and the multidrug-resistant *Candidozyma auris*. **Methods**: The antifungal activity of NMCA was assessed by analyzing fungal viability over time, ergosterol levels, and its interaction with fluconazole. Its antibiofilm activity was evaluated through biomass and metabolic activity measurements, together with the expression of genes involved in adhesion (*ALS3*, *ALS5*, *HWP1*) and membrane homeostasis (*ERG11*, *OLE1*). In addition, infected epithelial models were used to investigate epithelial damage, intracellular fungal burden, oxidative stress, and wound closure. **Results**: NMCA showed promising antifungal activity (MIC80 75 μg mL^−1^ against *C. albicans* and 100 µg mL^−1^ against *C. auris*), inducing a time-dependent reduction in fungal viability of about 4-log10 after 24 h. The compound also reduced ergosterol levels and showed synergistic interaction with fluconazole, as indicated by FICI values of 0.203 for *C. albicans* and 0.375 for *C. auris*. Moreover, NMCA markedly inhibited biofilm formation by reducing both biomass and metabolic activity up to approximately 80%, while modulating the expression of key adhesion- and membrane-related genes. Beyond its direct antifungal effects, NMCA reduced epithelial damage and intracellular fungal burden, attenuated oxidative stress, and significantly improved wound closure (reaching 76.26% and 90.46% closure in *C. albicans*- and *C. auris*-infected cells, respectively) in infected epithelial models. **Conclusions**: Although limited by the use of in vitro systems, these findings highlight the multifunctional profile of NMCA, which combines antifungal, antibiofilm, and tissue-protective activities. By simultaneously targeting pathogen viability, biofilm formation, and host cell integrity, NMCA appears to be a promising adjunctive candidate for the treatment of ocular fungal infections, where both pathogen eradication and tissue preservation are crucial for clinical outcomes.

## 1. Introduction

Fungal infections represent a growing global health challenge, contributing substantially to morbidity, mortality, and healthcare costs [1]. The rising incidence of invasive and localized mycoses is driven by the expanding population of immunocompromised and elderly individuals as well as the widespread use of medical devices that facilitate fungal colonization and biofilm development [2,3,4].

Among the various clinical manifestations of fungal diseases, infections affecting the eye are particularly challenging due to their potential to impair visual function and the limited therapeutic options currently available. Fungal pathogens can infect several ocular structures, including the eyelids, conjunctiva, lacrimal apparatus, sclera, cornea, and intraocular tissues. Among these conditions, fungal keratitis and endophthalmitis represent the most severe forms of ocular mycoses, as they can rapidly lead to irreversible visual impairment if not appropriately treated. The clinical management of these infections remains complex because diagnosis is often delayed and antifungal drugs may show limited penetration into ocular tissues [5,6].

Although *Candida albicans* is a commensal member of the human microbiota, alterations in host or environmental conditions can enhance its pathogenic potential. Biofilm formation is a major virulence factor that promotes persistence, antifungal tolerance, and chronic infection [7,8]. While *C. albicans* remains the leading cause of candidiasis and the predominant species associated with ocular candidiasis and fungal keratitis, recent epidemiological trends indicate an increasing prevalence of non-*albicans Candida* species. Among these, *Candidozyma auris* (*Candida auris*) has emerged as a multidrug-resistant opportunistic pathogen responsible for healthcare-associated outbreaks worldwide [9,10]. Although ocular infections caused by *C. auris* are relatively uncommon and mainly reported as sporadic cases or localized outbreaks, its global dissemination, environmental persistence, biofilm-forming ability, and reduced susceptibility to multiple antifungal agents make it an important target for the development and evaluation of novel antifungal strategies. 

*C. auris* has been reported in localized infections, including ocular involvement such as keratitis and endophthalmitis. Beyond its clinical relevance as an emerging pathogen, *C. auris* exhibits several characteristics that are highly pertinent to ocular infections, including robust biofilm formation, persistence on biological and abiotic surfaces, and reduced susceptibility to multiple antifungal agents. These features contribute to treatment failure and mirror some of the major challenges encountered in the management of fungal keratitis and device-associated ocular infections. Therefore, alongside *C. albicans*, *C. auris* represents a clinically relevant model for evaluating novel antifungal and antibiofilm strategies against a challenging multidrug-resistant Candida phenotype [11].

Therapeutic options for fungal infections remain limited to four main antifungal classes, with azoles representing the most widely used agents. Fluconazole (FLC), which targets lanosterol 14α-demethylase in the ergosterol biosynthesis pathway, is commonly prescribed due to its favorable pharmacological profile. However, the emergence of azole-resistant strains, particularly *C. auris*, underscores the urgent need for alternative or supportive therapeutic approaches [12,13]. In this context, drug repurposing and combination therapy have gained increasing attention as strategies to enhance antifungal efficacy while reducing the time and cost associated with new drug development. Tranilast, a clinically approved anti-inflammatory agent, and its derivatives may represent promising candidates due to their potential modulatory effects on inflammation, microbial invasion, and tissue repair.

**Figure 1:**
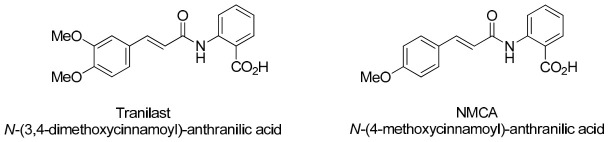
Structure of tranilast and *N*-(4-methoxycinnamoyl)-anthranilic acid (NMCA).

In our previous study [14], a biological screening of a series of Tranilast derivatives revealed that *N*-(4-methoxycinnamoyl)-anthranilic acid (NMCA; Figure 1) exhibited the lowest MIC values (75 μg/mL^−1^) against all three standard microorganisms tested, including *Candida albicans*. In addition to its superior antimicrobial activity, the compound displayed antioxidant, anti-inflammatory, and low cytotoxicity properties, while maintaining favorable selectivity toward mammalian cells. Given its consistent inhibitory activity against *Candida* spp. and its promising overall biological profile, NMCA was identified as the most suitable candidate for further investigation and was therefore selected for a comprehensive evaluation of its antifungal, antibiofilm, synergistic, and host-protective potential. The present study aims to investigate the antifungal and antibiofilm activity of NMCA, administered alone or in combination with a standard antifungal agent, as a supportive strategy for ocular fungal infections. Additionally, we evaluated its ability to inhibit epithelial cell invasion by *C. albicans* ATCC 90028 and *C. auris* DSM 21092 and to promote wound healing in infected epithelial monolayers.

## 2. Results

### 2.1. Antifungal Activity Against Candida Albicans and Non-Albicans Species 

The MIC_80_ and MFC of NMCA on *C. albicans* and *C. auris*. are shown in Table 1.

The two tested strains were susceptible to FLC with MIC of 1 µg mL^−1^ and 32 µg mL^−1^ for *C. albicans* and *C. auris* respectively. The antifungal effect of NMCA against the two strains showed that MIC values increased in a dose-dependent manner and that MFC values were higher than 200 µg mL^−1^.

To better investigate the impact of NMCA on the growth of C. *albicans* and *C. auris* over time, time-killing curves for two different doses were plotted using the colony counting method.

**Figure 2:**
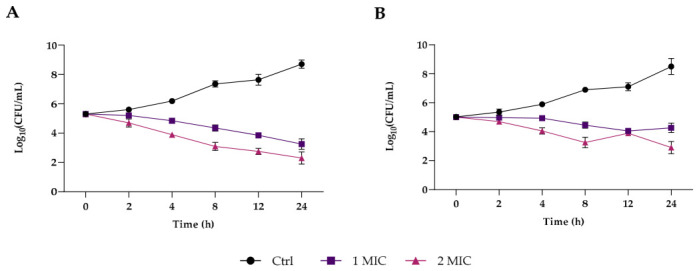
Time-kill curve of *C. albicans* (**A**) and *C. auris* (**B**) under the action of NMCA at concentrations of 1 MIC and 2 MIC.

As shown in Figure 2, NMCA decreased the viability of the *Candida* growth within 24 h compared with the untreated controls. The strongest effect was observed at the 2 MIC against *C. albicans*, where the treatment effectively inhibited fungal proliferation and significantly reduced the number of viable cells over time, resulting in an approximately 4-log_10_ decrease in CFU after 24 h. Similarly, for *C. auris*, a reduction in CFU was observed at 24 h, reaching approximately a 4-log_10_ decrease compared with the untreated control at concentration 2 MIC.

**Figure 3:**
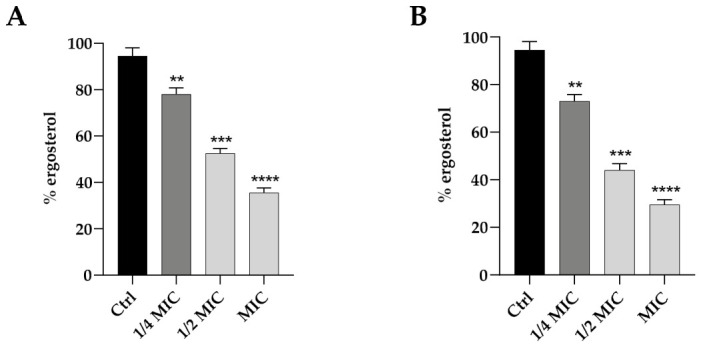
Ergosterol (μg mL^−1^) levels of (**A**) *C. albicans* and (**B**) *C. auris* not treated (Ctrl) and treated with NMCA at 1/4, 1/2 1× MIC. * indicated significant differences vs. control (** *p* < 0.01, *** *p* < 0.001, **** *p* < 0.00001, Dunnet’s test).

Ergosterol, the principal sterol component of the fungal cell membrane, plays a critical role in preserving membrane structure and function. By interacting with membrane phospholipids, it regulates permeability, fluidity, and the transport of molecules across the lipid bilayer. As shown in Figure 3, ergosterol levels were quantified in yeast cells treated with different concentrations of the NMCA. A significant reduction in ergosterol content was observed at higher concentrations; however, the decrease was more pronounced when the compound was administered at 1× MIC against both yeast cells.

### 2.2. Checkerboard Assay: NMCA Synergizes with FLC Against the Two Fungal Pathogens

We next evaluated the combined activity of the NMCA and FLC to determine whether their interaction could enhance antifungal efficacy and reduce the effective concentrations of both agents.

**Figure 4:**
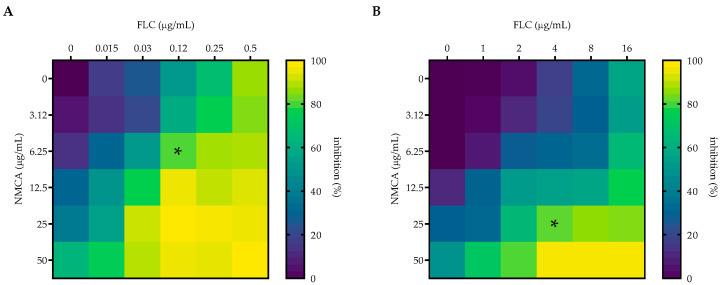
Checkerboard assay of interaction between NMCA and fluconazole (FLC) against *C. albicans* (**A**) and *C. auris* (**B**). * Indicated Fractional Inhibitory Concentration index (FICI).

As shown in Figure 4, the combination exhibited a clear synergistic antifungal effect, evidenced by a marked reduction in the MIC values of both compounds. For *C. albicans*, the MIC of the NMCA decreased 12-fold (NMCA/FLC = 6.25/0.12), whereas for *C. auris* a 4-fold reduction (NMCA/FLC = 25/4) was observed. In both species, the MIC of FLC was reduced 8-fold when used in combination.

The fractional inhibitory concentration index (FICI), calculated as an indicator of drug interaction, was 0.203 for *C. albicans* and 0.375 for *C. auris*. These values confirm a synergistic interaction in both cases, demonstrating that the concentrations required to inhibit fungal growth were substantially reduced when the two agents were administered together.

### 2.3. NMCA Exhibited Activity Against C. albicans and C. auris Biofilm Formation 

**Figure 5:**
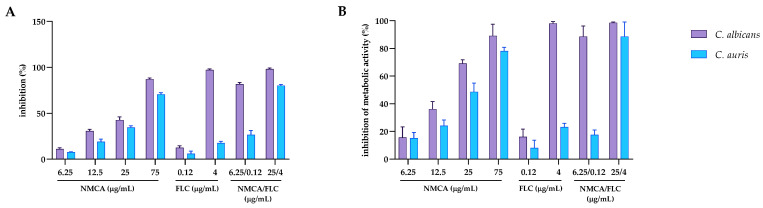
Effect of NMCA on biofilm formation by *C. albicans* and *C. auris.* (**A**) Total biofilm biomass quantified by crystal violet (CV) staining and expressed as percentage of biofilm inhibition relative to the untreated control. (**B**) Viable biofilm biomass expressed as percentage of inhibition of metabolic activity relative to the untreated control. Untreated biofilms grown in the absence of NMCA and FLC were used as controls and considered as 0% inhibition, corresponding to 100% biofilm biomass or metabolic activity.

The effect of different drug concentrations on biofilm formation was shown in Figure 5. Our study demonstrated that the NMCA significantly inhibited biofilm formation in both yeast strains at the highest concentration tested.

As shown in Figure 5A, NMCA reduced biofilm biomass in both *C. albicans* and *C. auris* in a concentration-dependent manner, reaching approximately 80% inhibition at the highest tested concentration. Similar inhibition values were obtained with the NMCA/FLC combinations at the FICI concentrations, 6.25/0.12 µg/mL for *C. albicans* and 25/4 µg/mL for *C. auris*. In agreement with the biomass data, XTT analysis showed a concentration-dependent decrease in biofilm metabolic activity, with the highest inhibition observed at the maximum NMCA concentration and at the FICI combinations (Figure 5B). Overall, the figure highlights both the dose-dependent effect of the NMCA and the enhanced efficacy of the drug combination strategy.

### 2.4. Effect of the Two Drugs on Gene Expression in C. albicans and C. auris

**Figure 6:**
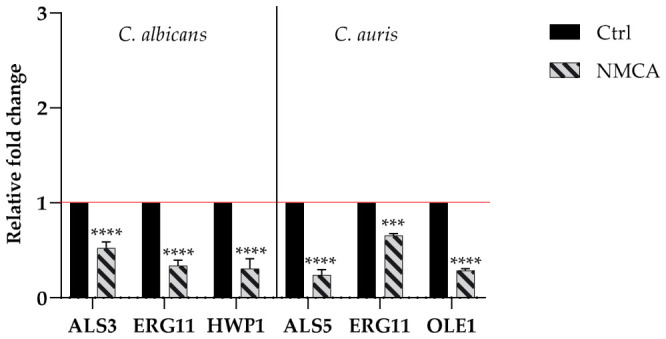
Relative expression of selected genes in *C. albicans* and *C. auris* following treatment with NMCA at concentration of 25 µg mL^−1^. The red line indicates the reference value of the untreated control, set at 1, used for the calculation of relative fold change. Asterisks indicate significant differences from own control *** *p* < 0.001, **** *p* < 0.00001, Tukey’s test).

Following the observation that the compound significantly impaired yeast biofilm formation on both strains *C. albicans* and *C. auris*, we evaluated their impact on the transcriptional regulation of key genes implicated in biofilm development. As reported in Figure 6, a significant downregulation of *OLE1*, *ERG11*, and *ALS5* was observed following both treatments. *OLE1* is involved in fatty acid desaturation and membrane fluidity, ERG11 in ergosterol biosynthesis, and *ALS5* in adhesion and biofilm formation. Their reduced expression indicates a potential impairment of lipid metabolism, membrane integrity, and biofilm-associated processes in *C. auris*. Likewise, gene expression analysis revealed a significant downregulation of *ALS3*, *HWP1*, and *ERG11* in treated *C. albicans* cells compared to the untreated control. *ALS3* encodes a cell surface adhesin involved in adhesion to host tissues and in the early stages of biofilm formation, while *HWP1* encodes hyphal wall protein 1, a key adhesin required for hyphal development and stable biofilm maturation. *ERG11* encodes lanosterol 14α-demethylase, an essential enzyme in the ergosterol biosynthetic pathway and the primary target of azole antifungals. The reduced expression of these genes suggests an impairment of adhesion-related processes, hyphal growth, and membrane sterol biosynthesis under the experimental conditions.

### 2.5. LDH Release

**Figure 7:**
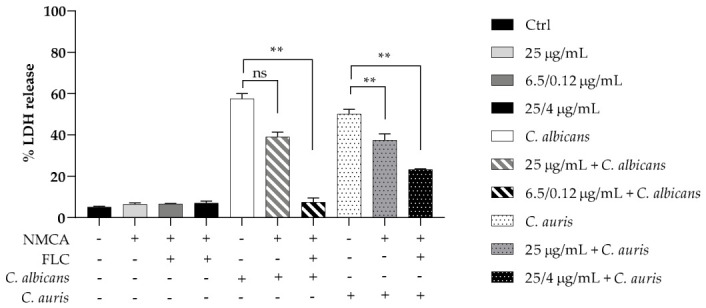
Spectroscopic determination of the release of LDH performed on HaCaT cells after incubation in the absence or in the presence of *Candida* spp. at a multiplicity of infection (MOI) of 5:1 and NMCA alone or in combination with FLC. Samples were examined using one-way ANOVA and Dunnet’s test. Asterisks (**) are used to indicate a considerable difference from own controls (*p* < 0.01, ns= not significant).

The cytotoxicity of both yeast strains in the presence of the NMCA was assessed using HaCaT cells. As shown in Figure 7, the NMCA alone did not exhibit cytotoxic effects. After 24 h of incubation, LDH release, used as a marker of membrane integrity, indicated that the tested concentration did not induce significant structural damage compared to untreated controls. In contrast, infection with *Candida* strains resulted in a marked increase in LDH release, reflecting substantial host cell damage. Co-treatment with the NMCA (25 μg mL^−1^) significantly reduced LDH release in infected cells. Although cytotoxicity was not completely abolished, the reduction in LDH levels indicates that the compound effectively mitigates *Candida*-induced cellular damage. This protective effect may be associated with its antifungal activity or its ability to interfere with pathogen-mediated mechanisms of host cell injury. Notably, LDH release was further decreased when the compound was administered at its FICI concentration in combination with FLC. This effect was observed for both strains and was more pronounced against *C. albicans*.

### 2.6. Evaluation of NMCA Invasion Capability

**Figure 8:**
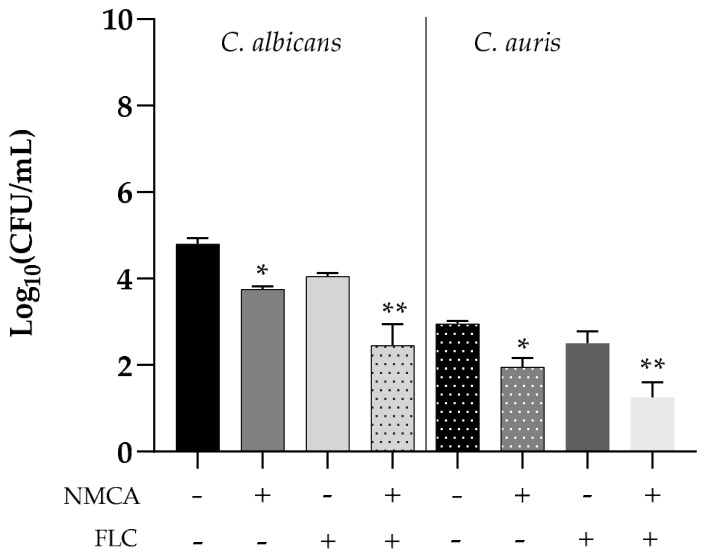
Inhibition of the invasion in HaCaT of *C. albicans*, and *C. auris* pre-treated with NMCA alone or with both FICI. Results were expressed in log_10_ (CFU mL^−1^) mean ± SD. Asterisks indicate significant difference from own controls (* *p* < 0.05, ** *p* < 0.01; Dunnett’s test).

Both *Candida* species were able to invade HaCaT cells following infection at MOI of 1:5. Assessment of invasive capacity revealed that pre-treatment with the NMCA, either alone or in combination with fluconazole, significantly reduced the invasiveness of both *C. albicans* and *C. auris* (Figure 8). The strongest effect was observed with the FICI combination, which resulted in an approximately 2-log reduction in invasion for both strains.

### 2.7. Evaluation of Intracellular Reactive Oxygen Species (ROS)

**Figure 9:**
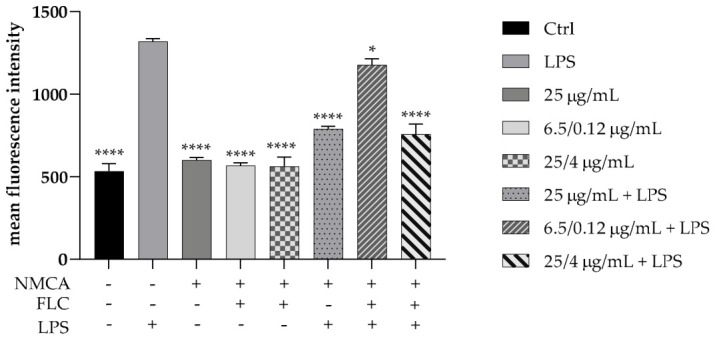
Intracellular reactive oxygen species (ROS) production in HaCaT cells stimulated with lipopolysaccharide (LPS) and treated with compound NMCA, alone or in combination with fluconazole (FLC). Asterisks indicate significant difference vs. LPS (* *p* < 0.05, **** *p* < 0.0001; Dunnett’s test).

ROS production was significantly reduced in LPS-stimulated HaCaT cells after 24 h of treatment with the NMCA. Exposure to 25 μg mL^−1^ of the compound, either alone or in combination with fluconazole (NMCA/FLC; 25/4 μg mL^−1^), resulted in a marked decrease in ROS levels (Figure 9). A reduction in ROS production was also observed when a lower FICI concentration (6.5/0.12 μg mL^−1^) was applied to LPS-stimulated cells, although the effect was less pronounced compared with the higher concentration treatments. 

### 2.8. Wound Healing and Scratch Assay

**Figure 10:**
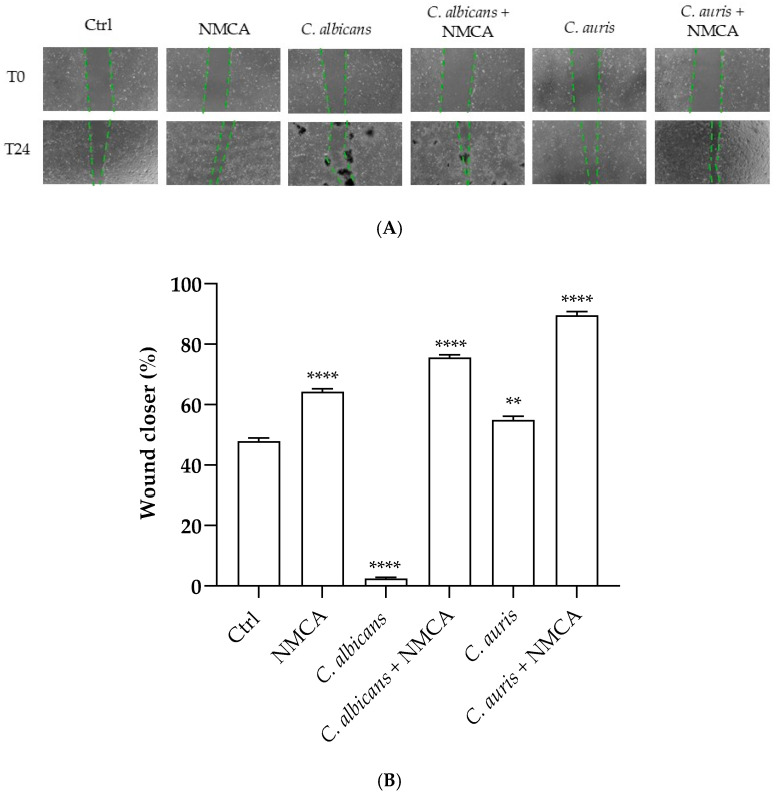
Effect of NMCA on wound closure in the presence of *Candida albicans* and *C. auris*. (**A**) Representative images of the wound healing assay at T0 and after 24 h (T24). (**B**) Percentage of wound closure after 24 h in control cells and in cells treated with NMCA, *C. albicans*, *C. auris*, or their combinations. Statistical significance is indicated by asterisks (** *p* < 0.01, **** *p* < 0.0001; Dunnett’s test).

To evaluate whether NMCA could promote keratinocyte proliferation and migration following mechanical injury, a wound healing (scratch) assay was performed. First, the optimal concentration of the compound was determined. Since no significant differences were observed among the tested concentrations, 25 μg mL^−1^ was selected for subsequent experiments, consistent with the conditions used in the other assays. At 0 h, microscopic observation confirmed a uniform scratch across all experimental groups, ensuring consistent wound generation. In the control group, cell migration into the wounded area occurred gradually; however, wound closure remained incomplete after 24 h of incubation. The scratch assay results showed that NMCA significantly enhanced the migratory capacity of HaCaT keratinocytes, promoting wound closure compared with the untreated control, with near-complete closure after 24 h (Figure 10A). Notably, a marked wound-healing effect was also observed in *C. albicans*-infected cells treated with the compound, reaching approximately 76.26% wound closure after 24 h compared with the infected, untreated control. Similarly, HaCaT cells infected with *C. auris* and treated with NMCA showed approximately 90.46% wound closure after 24 h (Figure 10B). Overall, these findings indicate that NMCA effectively promotes cell migration and wound closure, highlighting its strong potential as a wound-healing agent also in infected tissues.

## 3. Discussion

Fungal infections affecting the eye represent a significant clinical challenge because of their potential to compromise visual function and the limited availability of effective antifungal therapies. Among ocular mycoses, fungal keratitis and endophthalmitis are particularly severe conditions that can lead to permanent visual impairment if not promptly treated. The management of these infections is often complicated by delayed diagnosis, poor penetration of antifungal agents into ocular tissues, and the ability of fungal pathogens to form biofilms that increase resistance to therapy [15].

In our previous work [14], new hydroxylated analogues of tranilast were designed and synthesized to improve its solubility and bioavailability. These molecules were obtained through a green, single-step procedure, with good yields and without the need for chromatographic purification. The molecules were then evaluated for various biological activities, showing promising antimicrobial, antioxidant, anti-inflammatory, and antiproliferative effects. Some modified compounds exhibited enhanced efficacy and good selectivity, making them promising candidates for further pharmacological development.

In the present study, we investigated the antifungal and antibiofilm activity of NMCA against two major opportunistic fungal pathogens: *C. albicans* and *C. auris*. These species are increasingly associated with difficult-to-treat infections due to their ability to effectively colonize epithelial surfaces. Although the reference strain *C. albicans* ATCC 90028 was not originally isolated from an ocular source, it was chosen as the standard reference strain for antifungal susceptibility testing; furthermore, *C. albicans* remains the primary causative agent of ocular candidiasis, keratitis, and endogenous endophthalmitis.

While *C. albicans* is traditionally one of the most frequently isolated fungal species in human infections [14], the emergence of *C. auris* has raised global concern due to its multidrug-resistant phenotype and marked persistence in healthcare environments [15]. Although the susceptibility profile of *C. auris* DSM 21092 may not fully reflect the extreme resistance or virulence of specific clinical clades, this strain serves as a reliable reference model for exploratory investigations into this emerging threat, which is characterized by global dissemination, biofilm formation, and reduced sensitivity to conventional antifungals. More specifically, *C. auris* DSM 21092 was selected because it is a well-characterized reference strain that has been extensively employed in antifungal susceptibility studies and has also been used in our previous studies, allowing standardized comparisons and reproducible experimental conditions. Therefore, the findings obtained with this reference strain should not be directly generalized to all clinical *C. auris* isolates, particularly multidrug-resistant strains belonging to other geographic clades.

Several studies suggest that certain anti-inflammatory drugs exert antifungal and antibiofilm effects against *Candida* species. In particular it has been reported that diclofenac inhibits the growth of *C. tropicalis*, while drugs like piroxicam and aspirin show activity against *C. albicans*, including within biofilms. The antifungal effectiveness of anti-inflammatory drugs varies depending on the fungal species, strain, and environmental conditions such as pH. Ibuprofen, for example, shows strong activity against dermatophytes and *C. albicans*. Both steroidal and non-steroidal anti-inflammatory drugs, including dexamethasone, diclofenac, and piroxicam, effectively inhibit biofilm formation, as confirmed by experimental assays and microscopy [16,17,18]. Some authors have reported that dexamethasone, in particular, significantly reduces biofilm formation in *C. albicans*. This effect is linked to the downregulation of key genes (*ALS3*, *HWP1*, *EFG1*) involved in adhesion and biofilm structure, which weakens the fungus’s ability to form stable biofilms [19].

In the present study, NMCA exhibited significant antifungal activity against both *C. albicans* and *C. auris*, with MIC_80_ values of 75 and 100 µg mL^−1^, respectively. The progressive reduction in fungal viability observed over time suggests a time-dependent inhibitory effect, potentially linked to the marked decrease in cellular ergosterol levels (approximately 70% at the MIC). As ergosterol is a key component of the fungal membrane, essential for maintaining membrane integrity and fluidity, its depletion may compromise membrane stability and disrupt cellular homeostasis, ultimately impairing fungal survival. These findings indicate that NMCA may interfere with pathways involved in sterol biosynthesis or metabolism.

In addition to its intrinsic antifungal activity, NMCA demonstrated a strong synergistic interaction with fluconazole against both *Candida* species. Checkerboard assays revealed FICI values of 0.203 for *C. albicans* and 0.375 for *C. auris*, indicating a substantial enhancement of fluconazole efficacy and a marked reduction in its MIC values. Given the growing interest in combination therapies as a strategy to improve antifungal treatment and limit the emergence of resistance, these results highlight the potential of NMCA as an adjuvant agent. However, the translational relevance of this finding remains preliminary and should be validated using a larger collection of clinical isolates representative of different clades and resistance phenotypes.

Further investigations focusing on membrane permeability, intracellular fluconazole accumulation, and fungal stress-response pathways will be essential to clarify the molecular mechanisms underlying the observed synergistic effect. Therefore, although the synergistic interaction between NMCA and fluconazole was experimentally demonstrated by checkerboard analysis, the proposed mechanistic explanations remain hypothetical and require future experimental validation.

Biofilms represent a major virulence factor in *Candida* infections because they provide a protective environment that increases tolerance to antifungal treatments and host immune responses [20,21]. The reduction in both biofilm biomass and metabolic activity observed in this study, which reached a maximum inhibition of approximately 80%, indicates that NMCA may impair biofilm development. However, these assays provide indirect quantitative information on biofilm biomass and metabolic activity and do not directly assess biofilm architecture, thickness, or structural organization. Accordingly, it has been reported that also flavonoids (such as quercetin, catechin, and epigallocatechin gallate) are also promising synergistic agents when combined with fluconazole and are an effective antifungal agent against *C. albicans* biofilms [22,23,24]. 

Gene expression analyses further supported this observation, revealing the modulation of several genes involved in adhesion, biofilm formation, and membrane biosynthesis. Even though only a limited set of biofilm-related genes was examined, these findings suggest that the compounds may influence regulatory pathways controlling biofilm establishment and maturation. The ability to inhibit biofilm formation is particularly relevant in the context of ocular infections. Microbial biofilms can form on ocular surfaces and medical devices such as contact lenses, contributing to persistent infections and reduced treatment efficacy. Therefore, compounds capable of interfering with biofilm formation may represent valuable tools for improving the management of ocular fungal infections. The observed downregulation of *OLE1*, *ERG11*, *HWP1* and *ALS5/ALS3* suggests a coordinated modulation of pathways involved in membrane homeostasis and biofilm formation in *C. albicans* and *C. auris*. *OLE1*, which encodes a Δ9 fatty acid desaturase, plays a central role in the synthesis of monounsaturated fatty acids and in maintaining membrane fluidity. Reduced expression of this gene may alter lipid composition and compromise membrane adaptability under stress conditions. Similarly, *ERG11*, a key enzyme in ergosterol biosynthesis and the primary target of azole antifungals, is essential for maintaining membrane sterol integrity. Its downregulation may affect membrane structure and potentially influence antifungal susceptibility. The observed *ERG11* downregulation and reduction in ergosterol content suggest that NMCA may interfere with fungal membrane sterol homeostasis, thereby potentially enhancing susceptibility to fluconazole. However, the present findings do not allow us to determine whether NMCA directly targets the ergosterol biosynthetic pathway, facilitates fluconazole uptake through alterations in membrane permeability, or whether these effects represent secondary consequences of impaired fungal growth. Additional mechanistic studies will therefore be required to clarify the precise mode of action of NMCA.

In parallel, decreased expression of *ALS5/ALS3*, encoding adhesins implicated in surface attachment and biofilm development [25] and *HWP1* an important hypha-associated protein, essential for filamentation and biofilm formation [7], may contribute to reduced adhesive capacity and impaired biofilm formation. Given the well-established link between biofilm architecture, membrane composition, and antifungal tolerance, the concurrent downregulation of these genes may reflect a global impact on cellular processes that underpin persistence and resistance in both *Candida* strains. 

In addition, the potential impact of NMCA on host–pathogen interactions was investigated using epithelial cell models. Damage to epithelial tissues is a key event during ocular surface infections, where microbial invasion and inflammatory responses can lead to tissue destruction and delayed healing [26]. Our previous study demonstrated the low cytotoxicity of this compound in various cell lines. In the present study, treatment with NMCA significantly reduced cellular damage in *Candida*-infected cells, whereas infection with *Candida* alone induced measurable epithelial damage, as indicated by increased LDH release. Furthermore, the decreased number of intracellular fungal cells observed in treated cultures suggests that NMCA may interfere with mechanisms involved in fungal adhesion or invasion. In fact, limiting microbial invasion represents an important mechanism for controlling infection, as it prevents pathogens from penetrating host tissues and establishing deeper infections. 

These results indicate that NMCA exerts a protective effect on epithelial cells during fungal challenges. In addition to inhibiting fungal growth, the compound appears to interfere with mechanisms involved in host cell invasion. Notably, its combination with fluconazole enhances this effect, suggesting a synergistic interaction that further reduces the invasive capacity of *Candida* cells. This impairment of invasion may contribute to limiting host cell damage and supports the potential of NMCA as an adjunct strategy to improve antifungal therapy.

Regarding tranilast, several in vitro studies have consistently demonstrated its anti-inflammatory and antioxidant properties [14,27]. Since excessive production of ROS can amplify inflammatory responses and impair cellular integrity, the ability of NMCA to reduce ROS levels in LPS-stimulated epithelial cells may contribute to preserving cellular homeostasis and protecting epithelial tissues from inflammation-induced damage.

However, the relevance of these findings to fungal infections should be interpreted with caution. The antioxidant and cytoprotective effects observed in the LPS-stimulated HaCaT model may not fully reflect the host response elicited by *Candida* species. Therefore, the translational significance of these results in the context of fungal pathophysiology remains to be established and requires validation in *Candida*-stimulated epithelial models. Accordingly, the ROS findings should be interpreted as evidence of cytoprotective activity under experimentally induced inflammatory stress, rather than as direct evidence of antifungal immunomodulatory activity during *Candida* infection. Future studies employing direct *Candida*–host cell interaction systems, including ocular epithelial cell models, will be necessary to determine whether NMCA can effectively modulate infection-associated oxidative stress and inflammation. Such investigations will provide a more comprehensive assessment of the potential therapeutic relevance of NMCA in fungal infections.

Finally, wound-healing assays demonstrated that NMCA enhanced epithelial wound closure and promoted cell migration in both infected and non-infected HaCaT monolayers. Efficient epithelial repair is crucial for restoring tissue integrity following microbial injury, particularly at the ocular surface, where delayed re-epithelialization can prolong disease progression and increase the risk of complications. In this context, the ability of NMCA to accelerate wound closure suggests a potential role in supporting tissue repair processes.

However, the current experimental design does not allow us to determine whether this effect is primarily mediated by a direct stimulation of keratinocyte migration and/or proliferation, or indirectly through the reduction in fungal burden and the consequent attenuation of infection-induced cellular damage. Since NMCA significantly improved wound closure even in non-infected cells, a direct pro-reparative effect on epithelial cells cannot be excluded. Moreover, although in vitro models provide valuable mechanistic insights, they cannot fully reproduce the complexity of ocular infections in vivo, where tear film components, host immune responses, and biomechanical factors collectively influence microbial behavior, tissue damage, and therapeutic efficacy.

Despite the limitations inherent to in vitro models, which cannot fully recapitulate the complexity of ocular infections in vivo, the results of this study highlight the multifunctional properties of NMCA. In addition to its antifungal activity against *C. albicans* and *C. auris*, NMCA effectively inhibited biofilm formation, reduced epithelial damage, and promoted epithelial wound repair. Such a combination of antifungal, antibiofilm, cytoprotective, and pro-reparative effects may be particularly advantageous in the management of ocular infections, where successful treatment depends not only on pathogen eradication but also on the preservation and restoration of tissue integrity.

Nevertheless, these findings should be considered preliminary, as the exclusively in vitro nature of the study limits their direct clinical translation. Further investigations will be required to elucidate the mechanisms underlying the effects of NMCA on epithelial regeneration and host–pathogen interactions, as well as to validate its therapeutic potential in advanced cellular systems, ex vivo models, and clinically relevant in vivo models of fungal infection.

## 4. Materials and Methods

### 4.1. Tested Substance 

4-Methoxycinnamoyl-anthranilic acid was obtained through mixed carbonic anhydride activation of 4-methoxycinnamic acid with isobutyl chloroformate and triethylamine in dry acetone, followed by coupling with 2-aminobenzoic acid in the presence of an additional equivalent of triethylamine, under mild conditions. The product was isolated after crystallization (80% yield). The overall procedure followed the methodology previously reported in ref [28]. 

### 4.2. Yeast Strains

The standard *Candida albicans* strain obtained from the American Type Culture Collection (ATCC90028) and *Candidozyma auris* DSM 21092, formerly referred to as *Candida auris* were used in this study. They were maintained on Yeast Peptone Dextrose agar (YPD) and colonies were transferred to tubes containing sterile saline to obtain suspensions of 10^6^ cells mL^−1^ that were then diluted in Roswell Park Memorial Institute medium (RPMI 1640), supplemented with L glutamine, with pH adjusted to 7.4 using 0.165 mol L^−1^ of morpholinopropanesulfonic acid (MOPS) (Sigma Chemical Co., St. Louis, MO, USA), in accordance with the Clinical and Laboratory Standards Institute (CLSI) M27-A3 instructions [29].

### 4.3. Determination of Minimum Inhibitory Concentration (MIC) and Minimum Fungicidal Concentration (MFC)

The antifungal activity of NMCA was determined according to the CLSI M27-A3 by adding in 96-well microtiter plates 1 × 10^3^ CFU mL^−1^ yeast cells and 10 μL of the compound at final concentrations ranging from 10 to 200 μg mL^−1^. The plates were incubated at 30 °C for 24 h. The MIC was defined as the lowest compound concentration that inhibited the 80% of fungal growth. For MFC, 50 μL from each no turbid well identified during MIC determination was spread on YPD agar plates and incubated for 24 h at 30 °C. The MFC was defined as the lowest compound concentration at which no colony was observed on plates [30]. 

### 4.4. Time-Killing Assay Against C. albicans and C. auris

Time-kill experiments were conducted using the RPMI medium as the growth medium. Yeast cells were exposed to the compound at concentration of 1 MIC to 2 MIC as previously described [30]. Aliquots of 10 μL were taken at 0, 2, 4, 8, 12, and 24 h after exposure serially diluted and plated on YPD agar until 24 h. CFUs were calculated based on the average of triplicate plates. The results were shown in log_10_ (CFU mL^−1^). Non-treated cells were used as negative controls. All time-kill experiments were performed in three independent biological replicates, each including technical triplicates. Results are expressed as mean ± SD.

### 4.5. Determination of Ergosterol Content

To determine the effects of NMCA on ergosterol levels in *C. albicans* and *C. auris*, the ethanol-KOH method was used to extract the total sterols as described previously [31]. Cell concentration was adjusted to 1 × 10^6^ cells mL^−1^ and three different concentrations of compound (1/4 MIC, 1/2 MIC and 1 MIC) were added and subjected to incubation at 30 °C for a duration of 24 h. Fungal mass (20.0 mg) was collected, lysed with an ethanolic solution of potassium hydroxide (25% KOH), mixed for 1 min, and incubated in a water bath at 85 °C for 1 h. Sterols were extracted by adding a mixture of n-heptane (Sigma-Aldrich, Darmstadt, Germany) and distilled water (3:1), followed by vigorous homogenization for 3 min. The supernatant was collected and the ergosterol content was determined by spectrophotometry between 240 and 300 nm. A calibration curve using an ergosterol standard (Sigma-Aldrich) was constructed and used to calculate the amount of ergosterol (μg mL^−1^). 

The ergosterol content was calculated using the following equations:

$$\%\;Ergosterol=\left[\left(A_{281.5}/A_{290}\right)\times F_{sample\;weight}\right]-\left[\left(A_{230}/A_{518}\right)\times F_{sample\;weight}\right]\times 100$$

### 4.6. Checkerboard Assay

For the antifungal synergy studies, combinations of NMCA with fluconazole (FLC) were evaluated to determine potential synergistic interactions. A two-dimensional serial microdilution assay was performed in 96-well microplates, with NMCA serially diluted along the horizontal axis and fluconazole along the vertical axis, generating different concentration combinations. Each condition was tested in triplicate, including additional wells containing each compound alone. Subsequently, 100 µL of yeast cell suspension were added to each well, and the microplates were incubated at 37 °C for 24 h. Interactions were assessed based on the respective MIC values. Synergistic effects were expressed as the fractional inhibitory concentration index (FICI), calculated as follows:

FICI=(MIC of compound A in combination/MIC of compound A alone)+(MIC of compound B in combination/MIC of compound B alone).

A FICI ≤ 0.5 was interpreted as synergy; 0.5 < FICI ≤ 4 as no interaction; and FICI > 4 as antagonism.

### 4.7. Examination of Ability to Inhibit Biofilm Formation in Cultures of C. albicans and Non-albicans Candida

To evaluate the inhibition of biofilm formation we followed our previous protocol [5], yeast cell suspensions at a concentration of 1 × 10^6^ cells mL^−1^ in RPMI 1640 medium were added to 96-well plates containing different concentrations of the tested compound (ranging from 6.25 to 75 μg mL^−1^ and FICI combination (NMCA/FLC) of 6.25/0.12 μg mL^−1^ for *C. albicans* and 25/4 μg mL^−1^ for *C auris*). Plates were incubated at 37 °C for 24 h without shaking and subsequently washed three times with PBS. Biofilm formation was assessed using both crystal violet staining to quantify total biomass and the XTT assay to evaluate metabolic activity of viable cells. For crystal violet staining, biofilms were fixed with 200 μL of methanol for 15 min, stained with 200 μL of 0.2% crystal violet for 20 min, and washed with PBS. Subsequently, 200 μL of 33% acetic acid was added to each well to solubilize the stained biofilm for 30 min. Optical density was measured at 570 nm (OD570) using a microplate reader. Total biofilm biomass was calculated as follows:

$$Total\;biomass(\%)=(OD/OD_{0})\times 100$$

where OD_0_ represents the untreated control.

Metabolic activity was determined using the XTT [2,3-bis-(2-methoxy-4-nitro-5-sulfophenyl)-2H-tetrazolium-5-carboxanilide] reduction assay according to the manufacturer’s instructions. Briefly, 200 μL of XTT solution was added to each well and incubated in the dark for 1 h at 37 °C. Absorbance was measured at 450 nm (OD_450_) using a microplate reader. Metabolic activity was calculated as follows:

$$Metabolic\;activity\;(\%)\;=\;({OD}_{450\;control\;}-{OD}_{450\;treated})/{OD}_{450\;control}\;\times \;100$$

XTT assays were performed in three independent biological experiments, with each condition tested in triplicate.

Untreated biofilms, grown in the absence of NMCA and FLC, were used as controls and were considered as 100% biofilm biomass and metabolic activity. The percentage of inhibition was calculated relative to these untreated controls.

### 4.8. Detection of Biofilm Gene Expression by RT-qPCR

The total RNA was extracted from the NMCA (25 µg mL^−1^) and untreated biofilms of *C. albicans* and *C. auris* using Direct-zolTM RNA Miniprep Plus Kit (ZYMO RESEARCH, Irvine, CA, USA) according to the manufacturer’s instructions. The concentration and integrity of extracted RNA were assessed using a NanoDrop 2000 spectrophotometer (Thermo Fisher Scientific Inc., Waltham, MA, USA). An iScriptTM cDNA Synthesis Kit (BioRad, Milan, Italy) was used to retrotranscribe 1000 ng of RNA for each sample following manufacturer’s instructions. A total of 1 μL cDNA was used as a template in the reaction (final volume 10 μL) with 0.3 mM of each primer of the following biofilm-associated genes reported in Table S1 and 1× SensiFAST SYBR Green master mix (Meridiana Bioscience/Bioline, distributed by Aurogene Srl, Rome, Italy) for qRT-PCR, which was performed in AriaMx Real-Time PCR instrument (Agilent Technologies, Inc., Santa Clara, CA, USA). The relative expression levels were determined by quantitative RT-PCR using the 2^−ΔΔCt^ approach [32].

### 4.9. Cell Culture Conditions

The HaCaT human keratinocyte cell line (Cytion GmbH, Heidelberg, Germany) was used in this study. Cells were maintained in Dulbecco’s Modified Eagle Medium (DMEM; Biological Industries, Beit HaEmek, Israel), supplemented with 10% Fetal bovine serum (FBS), 1% non-essential amino acids, 1% glutamine, 100 U mL^−1^ penicillin, and 10 µg mL^−1^ streptomycin. All cultures were incubated under standard conditions (37 °C, 5% CO_2_).

### 4.10. Lactate Dehydrogenase (LDH) Release Assay

Cell damage induced by *C. albicans* and *C. auris* cells in HaCaT cells treated or not with compound NMCA at concentration of 25 μg mL^−1^ and both FICI (NMCA/FLC) concentrations was evaluated through the quantification of lactate dehydrogenase (LDH) released into the culture supernatant, using a commercial LDH assay kit (Sigma-Aldrich). Treated HaCaT cells monolayers were infected with the two yeast cells at Multiplicity-Of-Infection MOI 5:1 and incubated at 37 °C with 5% CO_2_ for 24 h. After incubation, supernatants were collected and LDH release was quantified spectrophotometrically. The percentage of damage was calculated following the manufacturer’s instructions.

### 4.11. Effect of NMCA on the Invasion Ability of Microbial Cells to HaCat

To investigate the effect of NMCA on the invasive ability of the two strains toward HaCaT cells, confluent monolayers were treated with NMCA at a concentration of 25 μg mL^−1^ and both FICI (NMCA/FLC) concentrations for 2 h at 37 °C in a humidified atmosphere containing 5% CO_2_. Following pre-treatment, yeast cells (MOI 5:1) were added to each well and incubated for an additional 2 h under the same conditions. To evaluate invasion, monolayers were further incubated for 2 h in DMEM supplemented with 250 µg mL^−1^ gentamicin sulfate (Sigma-Aldrich) to eliminate extracellular microorganisms. Cells were then lysed with 100 µL of Triton X-100, and the lysates were serially diluted and plated onto YPD agar plates to count CFUs after incubation at 37 °C for 24/48 h to determine the number of internalized microorganisms (CFU mL^−1^).

### 4.12. Assessment of Intracellular Reactive Oxygen Species (ROS)

The ROS levels were determined using a ROS assay kitwith the fluorescent probe 2′,7′-dichlorofluorescein diacetate (DCFH-DA) (Molecular Probes, Eugene, OR, USA). Briefly, HaCaT cells were exposed to different conditions: either stimulated with lipopolysaccharide (LPS) from *Salmonella typhimurium* (10 μg mL^−1^; Sigma-Aldrich, St. Louis, MO, USA), treated with modified NMCA at concentrations of 25 μg mL^−1^ and both FICI (NMCA/FLC) concentrations. Cells were cultured in 12-well plates under the conditions described above. After washing the monolayers with sterile PBS, cells were incubated with 10 mM DCFH-DA for 1 h at 37 °C in the dark. Fluorescence intensity (excitation at 488 nm and emission at 540 nm) was measured using a multimode microplate reader (Varioskan LUX, Thermo Fisher Scientific, USA). ROS measurements were performed in three independent biological experiments, each carried out in technical triplicate. Results are expressed as mean fluorescence intensity ± SD.

### 4.13. Cell Migration Assay-Wound Closure

HaCaT cells were plated at a density of 4 × 10^5^ cells/well in 12-well plates with 2.5 mL of medium and incubated for 24 h at 37 °C in a 5% CO_2_ atmosphere. Once confluency was reached, a scratch was introduced across each monolayer using a sterile 200 μL pipette tip. The monolayers were then infected with the two *Candida* cells at MOI of 5:1 and incubated for 2 h at 37 °C to allow fungal adhesion. Wells were then gently rinsed four times with serum-free DMEM to remove detached cells and fresh antibiotic-free DMEM containing NMCA (25 µg mL^−1^) was added to each well.

Scratch closure was monitored by imaging at 0 and 24 h of incubation using an inverted microscope equipped with a digital camera.

The percentage of wound closure was calculated as:

$$\mathrm{Wound}\;\mathrm{closure}\;(\%)=\frac{A_{0}-A_{24}}{A_{0}}\times 100$$

where A_0_ represents the initial wound area at 0 h and A_24_ the wound area after 24 h. The wound-healing assay was performed in three independent biological experiments. For each experimental condition, at least three fields per well were acquired and analyzed. Results are expressed as percentage of wound closure ± SD.

## 5. Conclusions

In conclusion, this study demonstrates that NMCA exhibits antifungal, antibiofilm, and epithelial-protective activities against *C. albicans* and *C. auris*. Beyond inhibiting fungal growth, the compound reduces biofilm formation, limits epithelial damage, and promotes wound healing in infected epithelial models. Overall, these findings highlight the potential of NMCAmay represent a valuable adjunctive or supportive therapeutic approach rather than a standalone fungicidal agent, particularly for fungal infections involving epithelial tissues, including the ocular surface.

Nevertheless, the translational relevance of these findings remains preliminary. Future studies should evaluate NMCA against a broader collection of clinical isolates representing different geographic clades and antifungal resistance profiles. In addition, validation in physiologically relevant in vivo models will be essential to confirm its efficacy, safety, and potential clinical applicability.

## Figures and Tables

**Table 1:** Minimum inhibitory concentrations (MIC) and minimum fungicidal concentrations (MFC) of tested substances.

	NMCA (µg mL^−1^)		FLC (µg mL^−1^)	
	MIC_80_	MFC	MIC	MFC
*C. albicans* ATCC 90028	75	>200	1	nd
*C. auris* DSM 21092	100	>200	32	nd

nd: not determined.

## Data Availability

The original contributions presented in this study are included in the article/Supplementary Materials. Further inquiries can be directed at the corresponding authors.

## Supplementary Materials

The following supporting information can be downloaded at https://www.mdpi.com/article/10.3390/antibiotics15060597/s1: Table S1: Primer sequences.

## Abbreviations

ALS3	Agglutinin-Like Sequence 3
ALS5	Agglutinin-Like Sequence 5
ANOVA	Analysis of Variance
ATCC	American Type Culture Collection
cDNA	Complementary DNA
CFU	Colony Forming Units
CLSI	Clinical and Laboratory Standards Institute
CO_2_	Carbon Dioxide
CV	Crystal Violet
DCFH-DA	2′,7′-Dichlorofluorescein Diacetate
DMEM	Dulbecco’s Modified Eagle Medium
DSM	Deutsche Sammlung von Mikroorganismen
ERG11	Lanosterol 14α-Demethylase Gene
FBS	Fetal Bovine Serum
FICI	Fractional Inhibitory Concentration Index
FLC	Fluconazole
HWP1	Hyphal Wall Protein 1
LDH	Lactate Dehydrogenase
LPS	Lipopolysaccharide
MFC	Minimum Fungicidal Concentration
MIC	Minimum Inhibitory Concentration
MIC80	Minimum Inhibitory Concentration Causing 80% Inhibition
MOI	Multiplicity of Infection
MOPS	Morpholinopropanesulfonic Acid
NMCA	*N*-(4-Methoxycinnamoyl)-Anthranilic Acid
OD	Optical Density
OLE1	Fatty Acid Desaturase Gene
PBS	Phosphate-Buffered Saline
RPMI	Roswell Park Memorial Institute Medium
ROS	Reactive Oxygen Species
RT-qPCR	Reverse Transcription Quantitative Polymerase Chain Reaction
XTT	2,3-bis-(2-Methoxy-4-Nitro-5-Sulfophenyl)-2H-Tetrazolium-5-Carboxanilide
YPD	Yeast Extract Peptone Dextrose Medium
