# Supplementary material for: Exploring the Antifungal, Antibiofilm, and Wound Healing In Vitro Properties of N-(4-Methoxycinnamoyl)-Anthranilic Acid as a Supportive Strategy for Ocular Fungal Infections
Source: Antibiotics (Basel). 2026 Jun 11;15(6):597. doi: 10.3390/antibiotics15060597 (PMC13295292; doi:10.3390/antibiotics15060597)
Supplement: Supplementary file 1 [file antibiotics-15-00597-s001.zip › antibiotics-4302885-supplementary.pdf]

Exploring the Antifungal, Antibiofilm, and Wound Healing In Vitro Properties of *N*-(4-Methoxycinnamoyl)-Anthranilic Acid as a Supportive Strategy for Ocular Fungal Infections

Francesco Petrillo [1,†], Annalisa Buonanno [2,†], Angela Maione [2,*], Luigi Longobardo [3], Michele Reibaldi [1], Emilia Galdiero [2], Armando Zarrelli [3,*] and Marco Guida [2]

[1] Department of Ophthalmology, "City of Health and Science" Hospital, 10126 Turin, Italy; francesco.petrillo@aocardarelli.it (F.P.); michele.reibaldi@unito.it (M.R.)
[2] Department of Biology, University of Naples Federico II, 80126 Naples, Italy; annalisa.buonanno@unina.it (A.B.); egaldier@unina.it (E.G.); marco.guida@unina.it (M.G.)
[3] Department of Chemical Science, University of Napoli Federico II, Via Cinthia 4, 80126 Napoli, Italy; luilongo@unina.it
* Correspondence: angela.maione@unina.it (A.M.); zarrelli@unina.it (A.Z.)
† These authors contributed equally to this work.

Table S1. Primer sequences

Gene acronym	Microorganism	**Sequence (5′ → 3′)**
ACT1	*C. albicans*	F: AGCCCAATCCAAAAGAGGTATT
		R: GCTTGGGTCAACAAAACTGG
ALS3	*C. albicans*	F: CTAATGCTGCTACTGATAATT
		R: CCTGAAATTGACATGTAGCA
ERG11	*C. albicans*	F: ATTGTTGAAACTGTCATTG
		R: CCCCTAATAATATACTGATCTG
HWP1	*C. albicans*	F: CAGCCACTGAAACACCAACT
		R: CAGAAGTAACAACAACAACACCAG
ACT1	*C. auris*	F: GAAGGAGATCACTGCTTTAGCC
		R: GAGCCACCAATCCACACAG
ERG11	*C. auris*	F: GTGCCCATCGTCTACAACCT
		R: TCTCCCACTCGATTTCTGCT
OLE1	*C. auris*	F: GACGGATTGGACACTGTCGAC
		R: CAGATCCTCGGCAAGCTGCTC
ALS5:	*C. auris*	F: CCTTCTGGATCGGACACAGT
		R: AGTTGTGGTGGAGGAACCAG
